# Characteristics and Stimulation Potential with BMP-2 and BMP-7 of Tenocyte-Like Cells Isolated from the Rotator Cuff of Female Donors

**DOI:** 10.1371/journal.pone.0067209

**Published:** 2013-06-25

**Authors:** Franka Klatte-Schulz, Stephan Pauly, Markus Scheibel, Stefan Greiner, Christian Gerhardt, Jelka Hartwig, Gerhard Schmidmaier, Britt Wildemann

**Affiliations:** 1 Julius Wolff Institute, Center for Musculoskeletal Surgery, Charité-Universitaetsmedizin Berlin, Berlin, Germany; 2 Berlin-Brandenburg Center for Regenerative Therapies, Charité-Universitaetsmedizin Berlin, Berlin, Germany; 3 Department Orthopädie, Unfallchirurgie und Paraplegiologie, Universitaetsklinikum Heidelberg, Heidelberg, Germany; University of Liverpool, United Kingdom

## Abstract

Tendon bone healing of the rotator cuff is often associated with non-healing or recurrent defects, which seems to be influenced by the patient’s age and sex. The present study aims to examine cellular biological characteristics of tenocyte-like cells that may contribute to this impaired rotator cuff healing. Moreover, a therapeutic approach using growth factors could possibly stimulate tendon bone healing. Therefore, our second aim was to identify patient groups who would particularly benefit from growth factor stimulation. Tenocyte-like cells isolated from supraspinatus tendons of female donors younger and older than 65 years of age were characterized with respect to different cellular biological parameters, such as cell density, cell count, marker expression, collagen-I protein synthesis, and stem cell potential. Furthermore, cells of the donor groups were stimulated with BMP-2 and BMP-7 (200 and 1000 ng/ml) in 3D-culture and analyzed for cell count, marker expression and collagen-I protein synthesis. Female donors older than 65 years of age showed significantly decreased cell count and collagen-I protein synthesis compared to cells from donors younger than 65 years. Cellular biological parameters including cell count, collagen-I and –III expression, and collagen-I protein synthesis of cells from both donor groups were stimulated with BMP-2 and BMP-7. The cells from donors older than 65 years revealed a decreased stimulation potential for cell count compared to the younger group. Cells from female donors older than 65 years of age showed inferior cellular biological characteristics. This may be one reason for a weaker healing potential observed in older female patients and should be taken into consideration for tendon bone healing of the rotator cuff.

## Introduction

Non-healing and recurrent defects are the most frequent complications following surgical reconstructions of the tendon bone unit of the rotator cuff [1]–[4]. The outcome after rotator cuff reconstructions depends on many different biological and clinical factors. Patient’s age has been shown to be highly correlated with tendon tears and recurrent defects [1], [5]–[8], while over the age of 65 the risk of a poor clinical outcome is strongly increased [1], [6]. The influence of sex on the healing outcome is controversial. Some clinical cohort studies have shown an influence of sex, with a higher failure rate after arthroscopic rotator cuff repair [9] or a higher disability of the shoulder, arm and hand (DASH) score and decreased strength in female patients [10]. However, other authors have found no association between the sex of the patients and the healing outcome [5], [7], [11]. It has been hypothesized that sex hormones such as estrogens may cause differences in healing capacities between men and women. Magnusson et al. reported that estrogens can influence the healing by influencing collagen synthesis in the tendon [12]. Furthermore, postoperative complications may be associated with “social” components of gender, with different personality traits, attitudes and behaviors potentially causing these differences [13], [14]. Additionally, different working activities of men and women have been investigated and suggested to influence shoulder disorders [14].

Differences in healing rates of rotator cuff tears between men and women have only been shown in epidemiological studies. However, to date no relationship between the cellular characteristics of tenocytes of the rotator cuff and the sex of the patient has been demonstrated. In a previous study, we demonstrated differences between tenocytes of rotator cuffs of young (average 45.3 years) and aged (average 72.3 years) male donors [15]. Cells differed with respect to their cell count and stem cell potential, with cells of aged donors showing inferior parameters. The same experimental set up was also performed in the present study for cells of female donors and results were discussed with previous findings to investigate sex-related differences.

In daily clinical practice rotator cuff disorders are treated in the same manner for different patient cohorts. However, as different healing rates in various donor populations may be associated with differing cellular characteristics, it may be useful to reconsider the uniform treatment of rotator cuff tears. Many in vivo and in vitro studies have demonstrated that the application of growth factors, such as bone morphogenetic protein (BMP)-2 and -7, in rotator cuff surgery may be a potential treatment option for an improved tendon bone healing. It has been reported that important cellular characteristics of tenocytes, such as cell proliferation and matrix production, can be stimulated with BMP-2 and BMP-7 [16]–[21]. Additionally, BMP-2 and BMP-7 have been found to increase tendon bone biomechanical strength during healing in several in vivo experiments [22]–[26]. In the present study, the stimulation potential of tenocyte-like cells (TLCs) of the rotator cuff from women younger or older than 65 years of age was investigated to allow for the development of more patient specific therapies.

## Methods

### Ethic Statement

The Ethic commission of the Charité-Universitaetsmedizin Berlin, Germany, authorized the use of tendon samples under anonymous conditions, that otherwise would be discarded (Ethic number: EA1/060/09).

### Tendon Material

Supraspinatus (SSP) tendon samples were taken from patients undergoing either arthroscopic surgery for rotator cuff repair or from open shoulder surgery for reasons such as hemiarthroplasty after humeral head fractures. The biopsies were obtained 3 to 5 mm from the torn proximal tendon edge according to a standardized protocol. All patients gave their written informed consent.

Several clinical studies have reported that patients over the age of 65 years have a higher risk of sustaining recurrent defects post rotator cuff surgery [1], [7], [11], [27]. Accordingly, in the present study the age cut-off was set at 65 years. The demographic data of the female donor groups younger and older than 65 years of age are listed in table 1.

**Table 1:** Demographic data of the donor groups.

Group	Mean age [years] (Range)	Mean muscle fatty infiltration (range)	Mean tendon retraction (range)	Mean tear size (range)
		MRI assessment [53]	MRI assessment, intraoperative diagnostics [54]	MRI assessment, intraoperative diagnostics [55]
**Female <65 years (N = 6)**	55.7 (50–60)	0.5 (0–1)	1.0 (0–2)	1.8 (1–2)
**Females >65 years (N = 6)**	68.2 (65–74)	0.8 (0–1)	1.2 (0–2)	2.0 (1–3)

### Analysis of Cell Density

TLCs were isolated from the SSP tendon samples as described previously by collagenase type CLS II digestion [15]. After 1 week of culture in normal growth medium (DMEM/Ham’s F12 with 10% fetal calf serum (FCS) and 1% penicillin/streptomycin, all Biochrom AG, Germany) with 3 medium changes per week, an Alamar Blue assay (Biozol, Germany) was performed according to the manufacturer to quantify the number of cells in the flask by a standard curve. The cell count was normalized to the weight of the tendon sample to assess an approximate cell density.

### Analysis of Cell Count Over 14 Days

A total of 2.5×10^3^ TLCs at passage 2 were seeded per well in a 48-well plate in triplicates and cell count was analyzed at day 1, 4, 7, and 14 with an Alamar Blue assay according to a previous study using a standard curve method [15]. The cell count of the TLCs at day 4, 7 and 14 was normalized to cell count of day 1 by subtraction.

### Gene Expression Analysis

At passage 2, RNA was isolated from the cells and cDNA was synthesized as described previously [15]. Cells were characterized by analyzing gene expression of collagen-I (Col-I), Col-II, Col-III, and osteocalcin. Furthermore, tendon-related genes like scleraxis, tenomodulin, and mohawk were analyzed, as well as the gene expression of decorin, transforming growth factor (TGF)-β1, TGF-β2, and TGF-β3. The housekeeping gene glyceraldehyde 3-phosphate dehydrogenase **(**GAPDH) served as an internal control (Primer sequences see table 2). The Real-Time PCR protocol is described under paragraph “validation of multipotent differentiation by Real-Time PCR”. The relative gene expression levels were calculated with the 2^-ΔCt^ method.

**Table 2:** Primer.

Gene	Sequence forward primer	Sequence reverse primer	Source	°C
**Housekeeping genes**				
GAPDH	CCACTCCTCCACCTTTGACG	CATGAGGTCCACCACCCTGT	Primer3	
RPL13	CCTGGAGGAGAAGAGGAAAGAGA	TTGAAGGACCTCTGTGTATTTGTCAA	Primer3	
**Characterization markers**				
Col-I	TGACCTCAAGATGTGCCACT	ACCAGACATGCCTCTTGTCC	Primer3	64
Col-III	GCTGGCATCAAAGGACATCG	TGTTACCTCGAGGCCCTGGT	Primer3	64
Scleraxis	QuantiTect Primer Assay Kit SCXB (Qiagen, Hilden, Germany)			60
TNMD	TTGAAGACCCACGAAGTAGA	ATGACATGGAGCACACTTTC	[56]	60
Mohawk	TGGTTTGCTAATGCAAGACG	CCTTCGTTCATGTGGGTTCT	Primer3	60
Decorin	CGCCTCATCTGAGGGAGCTT	TACTGGACCGGGTTGCTGAA	Primer3	64
TGF-β1	AAGGACCTCGGCTGGAAGTG	AGGGCCAGGACCTTGCTGTA	Primer3	64
TGF-β2	CAACAGCACCAGGGACTTGC	AGCACAAGCTGCCCACTGAG	Primer3	64
TGF-β3	CTGCTGGAGGAGATGCATGG	GGCAGACAGCCAGTTCGTTG	Primer3	64
**Adipogenic markers**				
PPARγ	TGCAGTGGGGATGTCTCATA	CAGCGGGAAGGACTTTATGT	Primer3	60
LPL	TCCGTGGCTACCTGTCATTT	ACATCCTGTCCCACCAGTTT	Primer3	60
FABP4	TCAGTGTGAATGGGGATGTG	CCACCAGTTTATCATCCTCTCG	Primer3	60
**Osteogenic markers**				
OC	CCCAGGCGCTACCTGTATCAA	CTGGAGAGGAGCAGAACTGG	Primer3	64
ALPL	GGAAATCTGTGGGCATTGTG	CCCTGATGTTATGCATGAGC	Primer3	60
Runx2	GCCCCCAAACAGTATCTTGA	GCCTGAAGTGAGGTTTTAGGC	Primer3	60
**Chondrogenic markers**				
ACAN	CCAGTGCACAGAGGGGTTTG	TCCGAGGGTGCCGTGAG	[57]	64
COMP	GCAACACGGACGAGGACAAG	CGCCATCACTGTCCTTCTGG	Primer3	64
Col-II	CGCACCTGCAGAGACCTGAA	TCTTCTTGGGAACGTTTGCTGG	Primer3	66

RPL13: ribosomal protein L13; TNMD: tenomodulin; PPARγ: peroxisome proliferator-activated receptor gamma; FABP4: fatty acid binding protein; LPL: lipoprotein lipase; OC: osteocalcin; ALPL: alkaline phosphatase tissue-nonspecific isozyme; Runx2: runt-related transcription factor 2, ACAN: aggrecan, COMP: cartilage oligomeric matrix protein.

### Analysis of Col-I Protein Synthesis

The Col-I protein synthesis was analyzed from the cell culture supernatant of day 14 of cell count analysis. The MicroVue C1CP EIA Kit (TecoMedical, Germany) was used according to the manufacturer. The level of Col-I protein synthesis was normalized to total protein content, which was analyzed with the Coomassie Plus™ protein assay (Thermo Fisher Scientific, Germany).

### Analysis of Stem Cell Phenotype

A total of 2.5–5×10^5^ vital cells in passage 1 were stained according to an established stem cell panel from the core unit of the Berlin-Brandenburg Center for Regenerative Therapies (BCRT). The cells were stained as follows: Live/Dead reagent, antibodies against CD29, CD44, CD73, CD90, CD105 and a negative mix consisting of CD11b, CD14, CD19, CD34 and CD45 for 25 min at 4°C (for further details see table 3). These antibodies were selected according to the study by Dominici et al. [28], who reported on the minimal stem cell criteria. Unstained cells as well as isotype controls were used as controls. After fixation, cells were measured with the BD FACS Canto II system (BD Biosciences, Germany) and FACS Diva software. The data was analyzed using FlowJo 8.8.6 software. The expression of surface markers related to stem cells as well as negative markers is an important characteristic for the stem cell potential of cells.

**Table 3:** Antibodies used for FACS analysis.

Antibody	Marker specification	Results	
		<65 years	>65 years
CD29 PE (BL)	Cell adhesion	+++	+++
CD44 PE/Cy7 (BL)	Hyaluronic acid receptor	+++	+++
CD73 APC (BL)	Mesenchymal, endothelial, epithelial marker	+++	+++
CD90 PerCP/Cy5.5 (BL)	Fibroblast and stromal cell marker	+++	+++
CD105 FITC (BL)	Mesenchymal cell marker	+++	+++
CD11b V450 (BD)	Leukocyte marker	−	−
CD14 V450 (BD)	Monocyte marker	−	−
CD19 V450 (BD)	B-cell marker	−	−
CD34 PB (BL)	Hematopoietic progenitor marker	−	−
CD45 V450 (BD)	Pan-leukocyte marker	−	−
PE mouse IgG1, κ isotype control (BL)		−	−
PE/Cy7 rat IgG2b, κ isotype control (BL)		−	−
APC mouse IgG1, κ isotype control (M)		−	−
PerCP/Cy5.5 mouse IgG1, κ isotype control (BD)		−	−
FITC mouse IgG1, κ isotype control (BD)		−	−
Live/Dead AF (I)			

BL: Biolegend, Uithoorn, Netherlands; BD: BD Biosciences; M: Miltenyi; I: Invitrogen.

PE: phycoerythrin; APC: allophycocyanin; PerCP: peridinin chlorophyll protein; FITC: fluorescein isothiocyanat; PB: Pacific Blue; AF: Aqua Fluorescent reactive dye.

Results: +++: >95% positive staining; −: <2% positive staining.

### Multipotent Differentiation

Cells with stem cell potential need to have the ability for multipotent differentiation, which was analyzed with the following method. Cells at passage 2 were seeded into a 24-well plate and cultured until confluence. Afterwards, cells were incubated in differentiation medium: osteogenic medium (500 µM L-ascorbic acid, 10 mM β-glycerophosphate and 100 nM dexamathasone in normal growth medium), adipogenic medium (1 µM dexamethasone, 1 µM insulin, 0,5 mM isobutyl-methylxanthine (IBMX), and 60 µM indomethacine in normal growth medium), or with normal growth medium (control). Cells were incubated in osteogenic or adipogenic induction medium for 3 weeks with a change of medium every 3–4 days. Formed calcium deposits were visualized using Alizarin Red S staining (0.5%, Sigma-Aldrich, in 0.5 M HCL, 10 min). For quantification, staining was solubilized with 5% SDS (Roth, Germany) in 0.5 M HCL for 5 min, and then measured at 405 nm against the blank (5% SDS in 0.5 M HCL). For validation of the osteogenic differentiation, TLCs were stained for alkaline phosphatase (ALP) exemplarily. Cells were fixed with formalin and incubated in ALP staining solution (0.06% Fast Blue Bb salt (Waldeck GmbH & Co KG, Germany), 0.01% naphtol-AS-MX-phosphate (Sigma-Aldrich), 0.5% dimethylformamide (Sigma-Aldrich), 2 mM magnesium chloride (Merck, Germany), 0.1 M tris-base (Sigma-Aldrich) in dH_2_O, pH 8.5) for 30 min at 37°C. For adipogenic differentiation, the lipid vacuoles in the cells were stained with 0.3% Oil Red O (Sigma-Aldrich) for 10 min. The background was cleared with 60% isopropanol, and the staining was solubilized with 100% isopropanol for 10 min and measured at 490 nm against the blank (100% isopropanol). The osteogenic and adipogenic differentiation was normalized to the staining of undifferentiated cells.

For chondrogenic differentiation 2.5×10^5^ vital cells were pelleted into 15 ml falcon tubes. One cell pellet was incubated with chondrogenic medium: 100 nM dexamethasone (Sigma-Aldrich), 175 nM L-ascorbic acid (Sigma-Aldrich), 40 µg/ml proline (Sigma-Aldrich), 100 µg/ml pyruvate (Roth), 6.25 µg/ml insulin-transferrin-sodium selenite supplement (ITS, Sigma-Aldrich), 1.25 mg/ml bovine serum albumin (BSA, Sigma-Aldrich), 5.35 mg/ml linolenacide (Sigma-Aldrich), 10 ng/ml TGF-β1 (R&D Systems GmbH, Germany) in normal growth medium, and 1 pellet with normal growth medium served as a control. Medium was changed every 3–4 days for 3 weeks. After fixation (4% PFA), cell pellets were paraffin embedded, 4 µm slices were taken and stained with 1% Alcian Blue solution (Sigma-Aldrich) for 30 min. Counterstaining was performed with 0.1% Kernechtrot (Waldeck GmbH & Co KG) in 5% aluminiumsulfate for 5 min. Alcian Blue staining for chondrogenic differentiation was exemplarily performed only for n = 1 TLC culture for each donor group.

### Validation of Multipotent Differentiation by Real-Time PCR

Individual stimulation experiments were additionally performed to validate the multipotent differentiation. The osteogenic and adipogenic differentiation was performed in triplicates and the chondrogenic differentiation in duplicates for 2 cultures per group (n = 4) and lineage specific markers were analyzed by Real-Time-PCR.

RNA was isolated from the osteogenic differentiated cells after 1 and 2 weeks of culture and from the adipogenic and chondrogenic differentiated cells after 3 weeks. For osteogenic and adipogenic differentiation RNA was directly isolated from the 24-well plates with the NucleoSpin RNA II Kit (Macherey Nagel, Germany). For chondrogenic differentiation 2 cell pellets from each donor were pooled and homogenized using peqGOLD TriFast (Peqlab, Germany) with the Precellys system (25× 1.4 mm ceramic pellets and 3× 2.8 mm ceramic pellets, Peqlab) at 5000 rpm for 30 s repeated 3 times. Using chloroform, RNA was extracted to the aqueous phase, which was afterwards diluted 1∶1 with 75% ethanol and transferred to the NucleoSpin RNA II columns to purify RNA according to the manufacturers manual.

100ng RNA were transcribed into cDNA with the qScript cDNA Supermix (Quanta BioSciences, USA) according the manufacturer’s instructions. Real-Time PCR was performed with the Realplex Mastercycler system (Eppendorf). 1.25 ng cDNA was used as PCR template. The Sybr Green mastermix was prepared with the following components for each well: 12.5 µl Sybr Green Supermix (Quanta BioSciences), 1 µl primer mix (10 µM, forward and reverse primer 1∶1), and 6.5 µl RNase/DNase-free water. 20 µl of the master mix was added to each well. Details for the primers used are listed in table 2.

After an initial denaturation step, the following Real-Time PCR amplification protocol was repeated for 40 cycles: 95°C for 15 s, annealing temperature for 45 s, and 72°C for 30 s. The protocol was finished with a melting curve program. The Real-Time PCR results were analyzed with the Realplex software (Eppendorf). Several housekeeping genes were tested, but were regulated with differentiation. The housekeeping gene ribosomal protein L13 (RPL13) showed the weakest regulation between differentiated and undifferentiated cells. Relative expression levels were normalized to RPL13 (2^−ΔCt^ method).

### Potential for Self Renewal

A total of 1000 TLCs in passage 2 were cultured for 11 days in a 10 cm petri dish with normal growth medium and a change of medium 3 times a week. The experiment was performed in triplicates. For quantification, colonies were stained with 1% methylene blue in boratbuffer/1% azure in dH_2_O (1∶1, Sigma-Aldrich) for 10 min. Pictures were taken and number and average size of the colonies (range: 1–10 mm^2^) were analyzed using an image analyzing system with an adaptive threshold (ImageJ 1.44i, Wayne Rasband, National Institute of Health, USA). The colony forming unit (CFU) assay allows the quantitative analysis of the self renewing capacity of TLCs, which is one important criterion for stem cells.

### Test for Relative Activity of BMP-2 and BMP-7

The mouse myogenic cell line C2C12 react with osteogenic differentiation after BMP-2 and BMP-7 stimulation and serve therefore as an established system for testing the relative activity of BMP-2 and BMP-7. The osteogenic differentiation was measured by an increase in ALP activity. C2C12 cells were seeded at a density of 5×10^4^ vital cells per well in a 24-well plate in triplicates. 5 hours after seeding cells were stimulated with 200 ng/ml or 1000 ng/ml rhBMP-2 (Wyeth, USA) or rhBMP-7 (R&D Systems GmbH, Germany) in DMEM supplemented with 1% FCS and 1% penicillin/streptomycin. After 3 days of incubation cell count was analyzed by Alamar Blue assay and subsequently cells were incubated with ALP substrate solution (0.13% 4-nitrophenyl phosphate disodium salt hexahydrate (Sigma-Aldrich), 50 mM glycine, 100 mM tris-base, 1 mM magnesium chloride in dH_2_O, pH 10.5) for 60 min and reaction was measured at 405 nm. The ALP activity after BMP-2 and BMP-7 stimulation was normalized to the cell count.

### Application of Growth Factors

A total of 2×10^4^ vital cells were seeded with a drop-on method into a macro porous collagen scaffold (size 85 mm^3^: 6 mm diameter × 3 mm height, Optimaix, Matricel, Germany) consisting of highly oriented porcine Col-I for 3D-culture. The remaining experimental set up is listed in table 4. TLCs were treated with 200 ng/ml or 1000 ng/ml rhBMP-2 or rhBMP-7 in DMEM/HAM’s F12 (1∶1) supplemented with 5% FCS and 1% penicillin/streptomycin.

**Table 4:** Experimental set up for growth factor application.

Day	Experimental procedure	Analysis
**−4**	Seeding in 3D-culture	
**−1**	Medium replacement (w/o FCS)	
**0**	Alamar Blue assay; Growth factor treatment	Cell count
**3**	Alamar Blue assay; Growth factor treatment	Cell count
**5**	Alamar Blue assay; Growth factor treatment	Cell count
**7**	Alamar Blue assay;RNA isolation, Real-Time PCRMicroVue C1CP EIA	Cell countGene expressionCol-I protein synthesis

Using Real-Time PCR, relative gene expression levels of Col-I, -II, -III and osteocalcin were normalized to GAPDH and to the untreated control, and calculated using the 2^−ΔΔCt^ method [29]. The Col-I protein synthesis was analyzed by MicroVue C1CP EIA Kit (TecoMedical) and normalized to the total protein content measured with the Coomassie Plus™ protein assay according to the manufacturer.

### Statistics

Statistics were performed for n = 18 values (N = 6 donors per group in triplicates) for each donor group. For Real-Time PCR analysis, RNA of triplicates was pooled (n = 6). The results are always given as median with 25 and 75 percentiles. Statistical analysis was performed using SPSS 20 (IBM, USA). Significant differences between all groups for the stimulation potential were analyzed with the Kruskal-Wallis test. The Mann-Whitney U test was performed to compare the female groups younger and older than 65 years of age or the different concentration groups with the untreated control. The level of significance was set at p<0.05 and adjusted with the Bonferroni-Holm correction. For the analysis of stimulation potential an additional level of significance was investigated to indicate high significant values (p≤0.001).

## Results

### Cell Biology

**Figure 1:**
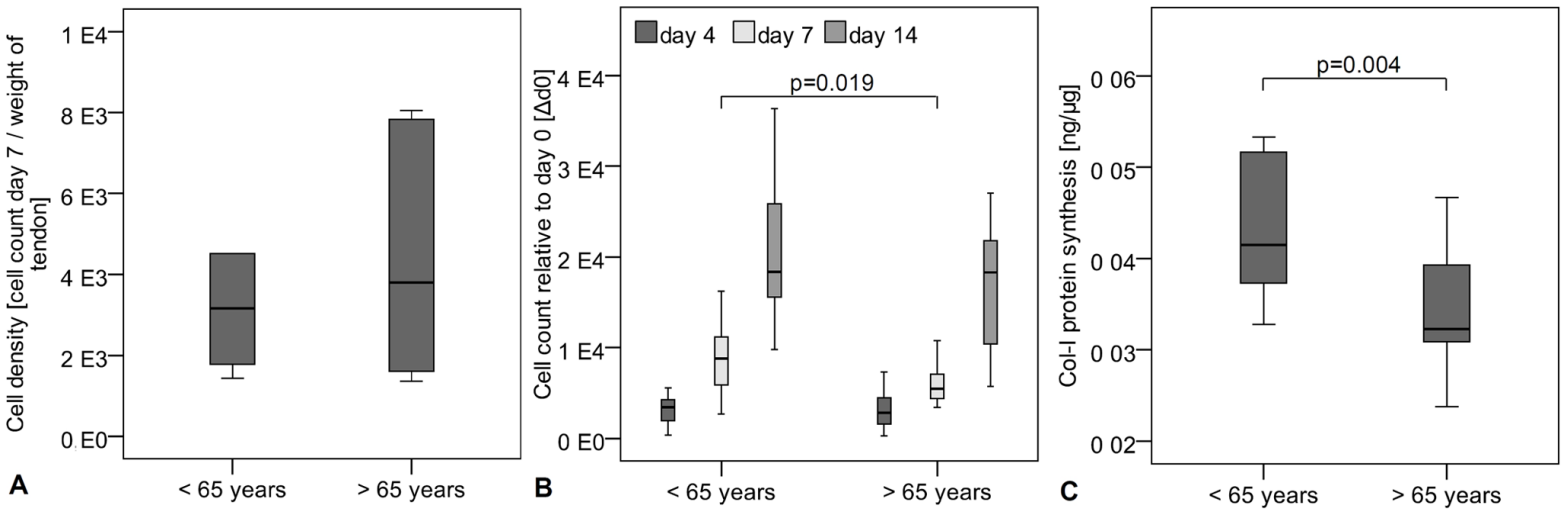
Cellular biological characteristics of TLCs of female donors younger and older than 65 years of age. **A** Cell density, measured by cell count 7 days after isolation relative to the weight of the tendon biopsy, showed no significant differences between the groups. **B** Cell count measured over 14 days and relative to day 1 after seeding was significantly decreased in TLCs of females >65 years compared to females <65 years. **C** Col-I protein synthesis relative to total protein content was significantly increased in female cells younger than 65 years of age.

**Figure 2:**
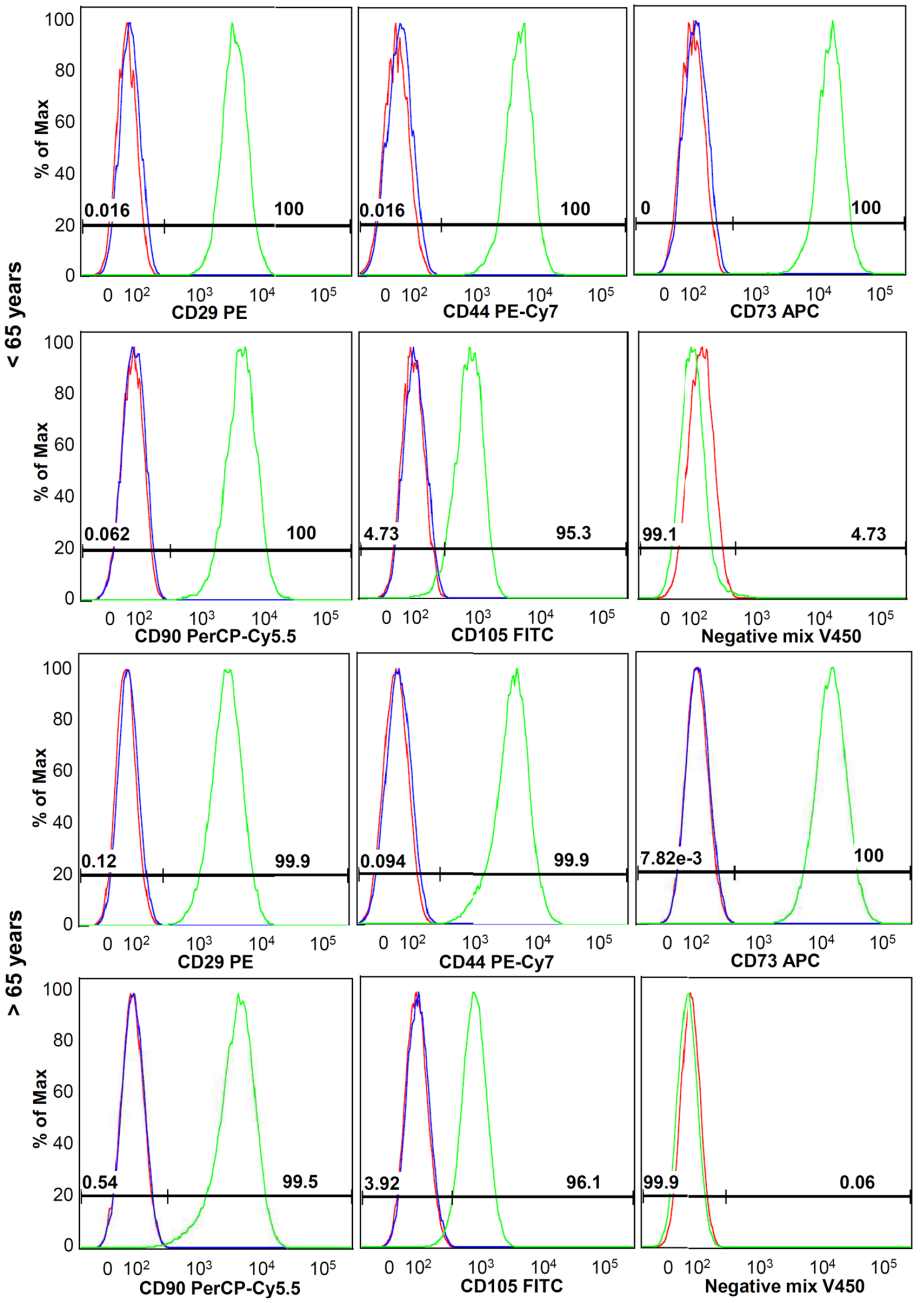
Representative histograms of FACS analysis of female donors </>65 years. Antibodies against stem cell markers as CD29, CD44, CD73, CD90 and CD105 revealed an overall more than 95% positive staining. Negative markers were found negative in more than 99% of cases. Green histograms represent stained cells, blue histograms represent isotype controls and red histograms show unstained cells.

**Figure 3:**
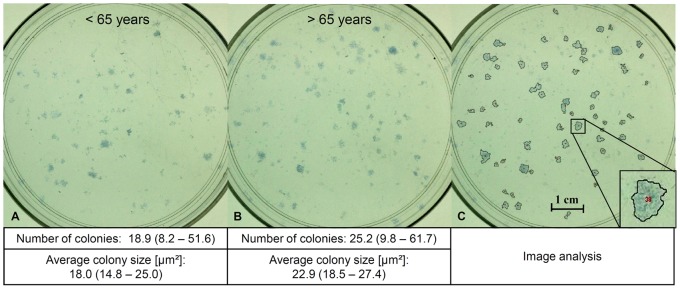
Representative images of CFU assay. Blue colonies formed from 1000 TLCs seeded on a 10 cm petri dish after methylen blue/azure staining from female TLCs. **A** <65 years, and **B** >65 years, **C** marked cell colonies after image analysis.

**Figure 4:**
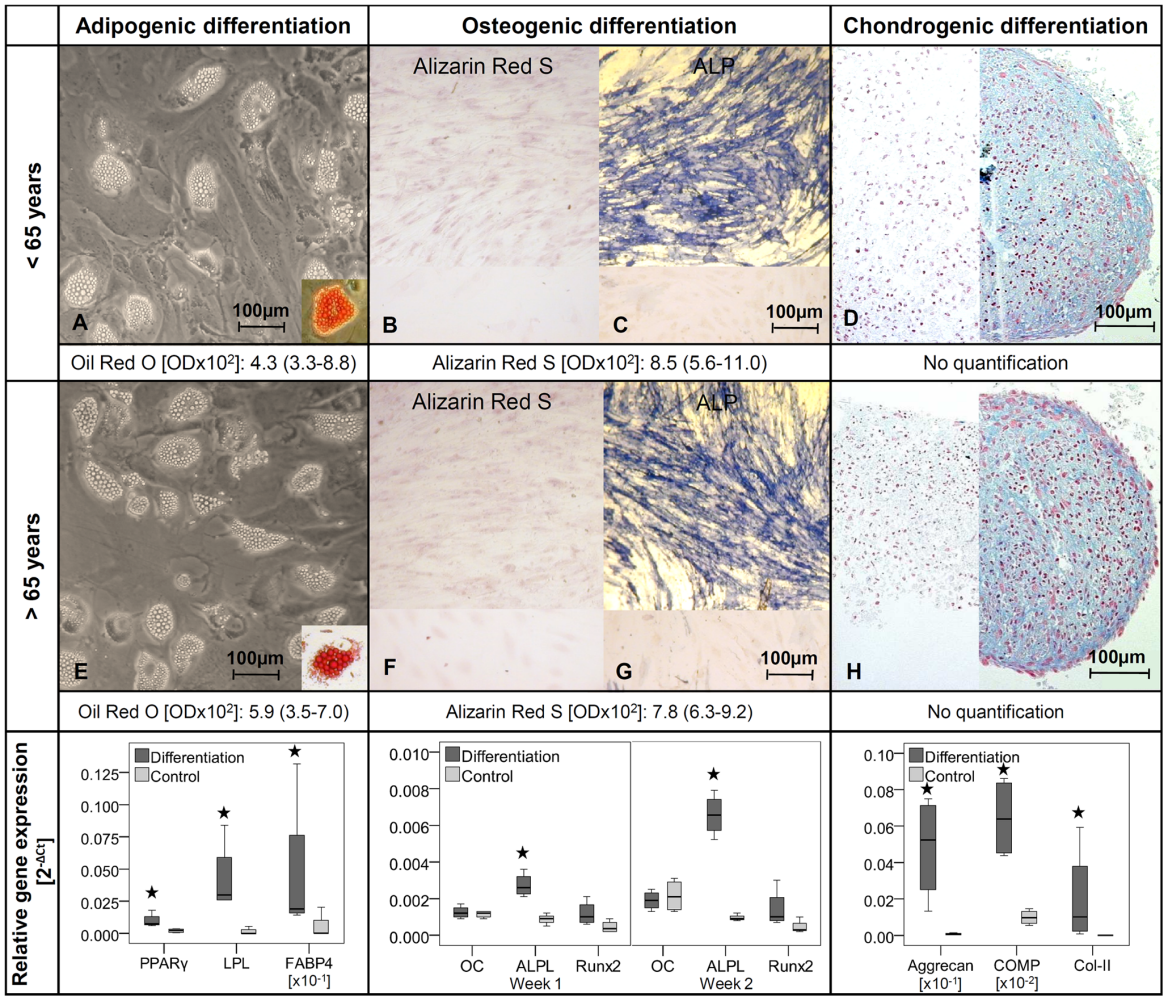
Multipotent differentiation potential of female TLCs. Representative images of multipotent differentiation of TLCs of female donors younger (A–D) and older (E–H) than 65 years. **A and E** Adipogenic differentiated cells showed lipid vacuoles, which were stained red after Oil Red O staining (cut-out). **B and F** Alizarin Red S staining after osteogenic differentiation was relatively weak in TLCs compared to the undifferentiated control cells (bottom). **C and G** ALP staining revealed a strong blue color in osteogenic differentiated TLCs compared to undifferentiated cells (bottom) **D and H** Blue stained chondrogenic differentiated cell pellets after Alcian Blue staining (right) versus control cell pellets (left). **Relative gene expression** of lineage specific markers was upregulated in the differentiated cells compared to undifferentiated control cells (p = 0.029).

No significant differences between female donors younger and older than 65 years of age were observed regarding cell density of the tendon samples (Figure 1A). Cell proliferation was reduced in the cells from donors <65 years, as seen in the lower cell count at day 7 (Figure 1B). The Col-I protein synthesis was significantly increased in TLCs of female donors younger than 65 years of age compared to the older group (Figure 1C). The TLCs of both donor groups expressed Col-I and Col-III, the most important collagens in the tendon. They expressed low amounts of osteocalcin but no Col-II. Tendon-related genes like scleraxis, and mohawk were also expressed in the cells, as well as decorin, TGF-β1, -β2, and -β3. Tenomodulin expression was not present in the TLCs. No significant differences in the expression profile were detected between both donor groups (Table 5). A portion of TLCs revealed a stem cell potential, as was evident by their expression of stem cell markers, potency for self renewal, and multipotent differentiation. Cells were overall more than 95% positive for the markers CD29, CD44, CD73, CD90 and CD105, but more than 98% negative for the markers CD11b, CD14, CD19, CD34 and CD45, without differences between the groups (Table 3, Figure 2). With respect to the potency for self renewal, no differences were found between the groups, while 1.9% (0.8–5.2%) of the cells of donors <65 years and 2.5% (1.0–6.2%) of the cells of donors >65 years were able to form adherent cell colonies (Figure 3). A portion of cells differentiated into an adipogenic, osteogenic and chondrogenic phenotype (Figure 4). However, the osteogenic Alizarin Red S staining as well as the chondrogenic Alcian Blue staining was relatively weak in both donor groups. The ALP staining was positive in cells differentiated to the osteogenic direction. Gene expression analysis of osteogenic differentiated cells revealed an increased expression of the differentiation marker alkaline phosphatase tissue-nonspecific isozyme (ALPL) after 1 and 2 weeks compared to undifferentiated cells. The expression of runt-related transcription factor 2 (Run×2) was upregulated in differentiated cells after 1 and 2 weeks compared to controls, however without significant differences (p = 0.114). The osteocalcin expression was not distinctly different in cells incubated in osteogenic or normal growth medium. TLCs, which were differentiated into the adipogenic direction showed an increased expression of adipogenic markers like peroxisome proliferator-activated receptor gamma (PPARγ), lipoprotein lipase (LPL) and fatty acid binding protein 4 (FABP4). In the chondrogenic differentiated TLC pellets a strong upregulation of aggrecan and cartilage oligomeric matrix protein (COMP) expression was found. Furthermore, cell pellets incubated with chondrogenic induction medium expressed Col-II, while no Col-II expression was present in pellets cultured with normal growth medium (Figure 4). The quantification of adipogenic and osteogenic differentiation by solubilizing of the stainings revealed no significant differences between the TLCs of both donor groups.

**Table 5:** Relative gene expression of TLCs of female donors.

Relative gene expression normalized to GAPDH [2^−ΔCt^ ] (median with 25–75 percentile)	Female<65 years	Female>65 years
**Col-I**	2.8 (2.4–3.3)	2.7 (2.2–3.3)
**Col-II**	–	–
**Col-III**	0.3 (0.2–0.8)	0.3 (0.2–0.5)
**Osteocalcin ** [<bold>10<sup>−4</sup></bold>]	5.5 (4.1–6.6)	5.9 (5.8–8.8)
**Scleraxis ** [<bold>10<sup>−3</sup></bold>]	2.5 (1.5–5.9)	2.3 (1.8–2.7)
**Tenomodulin**	–	–
**Mohawk ** [<bold>10<sup>−2</sup></bold>]	1.5 (0.9–3.2)	0.8 (0.7–2.1)
**Decorin**	0.2 (0.1–0.2)	0.2 (0.1–0.2)
**TGF- β1 ** [<bold>10<sup>−2</sup></bold>]	5.1 (3.7–5.8)	4.0 (3.3–4.9)
**TGF-β2 ** [<bold>10<sup>−4</sup></bold>]	2.1 (1.3–4.0)	1.8 (1.0–3.5)
**TGF-β3 ** [<bold>10<sup>−4</sup></bold>]	8.4 (4.7–11.4)	7.0 (4.7–9.3)

### Stimulation Potential

The relative activity of BMP-2 and BMP-7, tested by ALP activity normalized to cell count in C2C12 cells, were comparable between both factors at a concentration of 1000 ng/ml (BMP-2∶2.7 (2.5–2.8), BMP-7∶3.0 (2.7–3.1). At a concentration of 200 ng/ml ALP inducing activity of BMP-2 was stronger compared to the same concentration of BMP-7 (BMP-2∶2.2 (2.0–2.5), BMP-7∶0.6 (0.6–0.7)).

**Figure 5:**
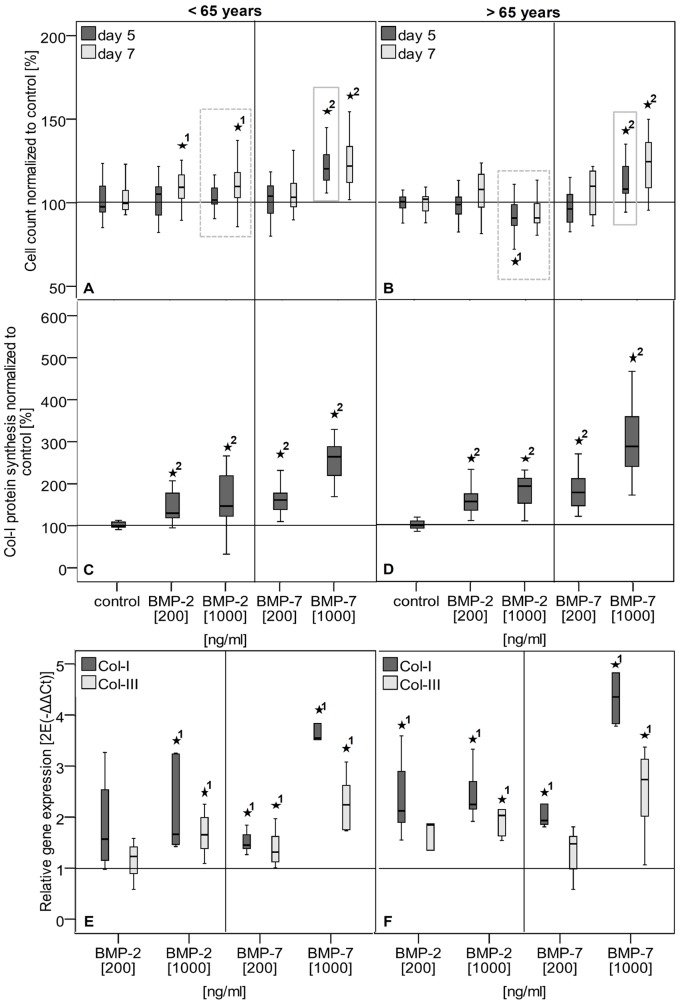
Stimulation potential of TLCs of female donors younger and older than 65 years of age. **A–B** Cell count of TLCs of female donors younger (A) and older (B) than 65 years was measured by Alamar Blue assay and given as percentage relative to untreated controls. BMP-2 application increased cell count at day 7 at the low and high concentration in cells of donors younger than 65 years. High BMP-2 concentration significantly decreased cell count at day 5 in TLCs of donors older than 65 years of age. Application of BMP-7 enhanced cell count at the high concentration at day 5 and 7 in cells of both donor groups. Gray boxes in graphs indicate significant differences between donors </>65 years, while older donors showed a decreased stimulated cell count. **C–D** Col-I protein synthesis in cell culture supernatant of day 7 after growth factor application of TLCs of female donors younger (C) and older (D) than 65 years of age. Col-I synthesis was calculated relative to total protein and given as percentage to the untreated control. The BMP-2 and BMP-7 treatment of TLCs of both donor groups significantly increased Col-I protein synthesis at all concentrations. **E–F** Col-I and Col-III gene expression after growth factor application of TLCs of female donors younger (E) and older (F) than 65 years. Gene expression is given as 2^−ΔΔCt^ and was normalized to the untreated control. The Col-I and Col-III expression was significantly increased by high BMP-2 and both BMP-7 concentrations in the female group<65 years. In cells of females >65 years both factors increased the Col-I expression, but Col-III expression was only increase by high concentrations of BMP-2 and BMP-7. The asterisks (*) mark significant differences to the untreated control. The numbers give details for the p-value: 1: p≤0.05; 2: p≤0.001.

The application of BMP-2 to TLCs of donors younger and older than 65 years of age in the 3D-culture slightly increased the cell count at day seven in cells of females younger than 65 years of age, but not in cells of females older than 65 years. With application of BMP-7, cell count was increased at the high concentration at days 5 and 7 in both donor groups (Figure 5A). A significantly different stimulation potential between cells of female donors younger and older than 65 years of age was found, while the stimulation of cell count was weaker in the older female group compared to the younger female group at high BMP-2 and BMP-7 concentrations (Figure 5A, gray boxes). The Col-I expression and protein synthesis was increased in TLCs of both groups after application of BMP-2 and BMP-7 at both concentrations, while the treatment of the cells with BMP-7 had a stronger effect than that with BMP-2. The BMP-2 stimulation increased the Col-III expression in the TLCs at the high concentration in both donors groups (Figure 5B). BMP-7 additionally led to an increased Col-III expression in the low concentration in cells of females younger than 65 years of age (Figure 5C).

## Discussion

The purpose of this study was to characterize TLCs isolated from SSP tendons of female donors of 2 different age groups to investigate differences in biological characteristics of the cell and stimulation potential with growth factors. Expanding upon our previous study investigating the effect of age on cells of male donors [15], the current study analyzed cells of female donors younger and older than 65 years of age. The results from female donors are also compared to previously published data from male donors to investigate additionally sex-associated differences. These cellular differences may be a reason for an inferior healing potential observed after rotator cuff tears in older or female patients.

Consistent with our previous study investigating age-related differences in male TLCs, the present study demonstrates that cells of older female donors have inferior cell biological characteristics compared to the younger female group. Cells of female donors older than 65 years of age had a lower cell count and Col-I protein synthesis. In comparison to our previous study, the age-related differences in the female TLCs were not as pronounced as those observed in males, but this may be explained by a lower mean age difference among female donors (15 years) compared to the male donors (25 years).

TLCs of female donors younger and older than 65 years of age showed a typical gene expression profile for tenocytes; cells expressed Col-I and Col-III the most important collagens in the tendon. The tendon related transcription factor scleraxis and the mohawk homeobox gene, which is a regulator in tendon development [30], were also expressed in the TLCs. Furthermore, decorin, the most important proteoglycan in the tendon [31], as well as the 3 TGF-β isoforms were expressed in the cells. Tenomodulin expression was not present in the TLCs. We reported previously that TLCs at passage 0 or 1 have a very weak tenomodulin expression [32]. This time mRNA expression from cells at passage 2 was analyzed, without measurable tenomodulin expression. Also other authors showed that tenomodulin expression is reduced in cells cultured 2 dimensionally and with higher passages [33], [34]. As negative control, no Col-II and low amounts of osteocalcin were expressed.

When comparing the results of female donors to the cell biological characteristics of male donors from the previous study sex-related differences became apparent. Cells of female donors older than 65 years of age showed less cell count, Col-I protein synthesis, and potential for self renewal, compared to the TLCs of older male donors. Only a direct comparison of the older female and older male group was meaningful, due to a comparable mean age of the groups. The cell count at day 4 and 14 in older female TLCs was 2793 and 18303, respectively, lower than in older male cells, 4648 and 25140 (p = 0.021/0.025). Also the Col-I protein synthesis was lower with 0.027 ng Col-I per µg total protein in female cells compared to 0.039 ng Col-I per µg total protein in older male TLCs (p = 0.012). Additionally, only 3.6% of TLCs of female donors were able to form adherent cell colonies, while 7% of older male donors formed colonies (p = 0.007). A study on human muscle derived stem cells also revealed differences between male and female cells [35]. The authors found increased cell count and differentiation rates, as well as lower apoptosis rates after oxidative stress injury in human female cells. Fossett et al. reported that synovial fat pad derived mesenchymal stem cells (MSCs) of female donors tend to show an increased proliferation rate and surface marker expression compared to male cells [36]. However, this correlation was not statistically significant. Another study on human MSCs from bone marrow revealed no significant differences between male and female donors for cellular characteristics including CFU potential, single cell cloning efficiency, generation time and multipotent differentiation [37]. Most of these studies indicate that female cells may be stronger or more resilient than cells from men, which is in contrast to our findings. However, differences may be a result of different cell types used, as these studies use a variety of cells from mesenchymal precursors versus specialized cells of the musculoskeletal system.

The analysis of stem cell phenotype revealed a more than 95% expression of surface markers related to stem cells like the cell adhesion molecule CD29, the hyaluronic acid receptor CD44, the mesenchymal cell markers CD73 and CD105, and the fibroblast marker CD90. The negative markers for leukocytes CD11b and CD45, the monocyte marker CD14, the B-cell marker CD19, as well as the hematopoietic progenitor marker CD34 were not expressed on the TLCs. This result is comparable to other studies on adult cell cultures of the anterior cruciate ligament (ACL) [38], [39] and hamstring tendon [40]. Steinert et al. reported that the isolation method, either by collagenase digestion or explants migration, has no influence on the expression of these respective surface markers [38].

The ability of cells to differentiate into the adipogenic, osteogenic, and chondrogenic direction is one of the minimal stem cell criteria investigated by Dominici et al. [28]. A better differentiation potential of the cells may therefore be linked to a greater number of tendon stem cells in the culture. However, the osteogenic Alizarin Red S staining, as well as the chondrogenic Alcian Blue staining was relatively weak in cells of both donor groups. A reason may be that the analyzed TLCs are a mixture of tenocytes and some tendon stem cells. A weaker staining or differentiation compared to pure MSC cultures was therefore expected. Real-Time PCR analysis of differentiation markers were additionally performed to prove differentiation towards multiple directions. In adipogenic differentiated TLCs the upregulation of lineage specific markers PPARγ, LPL and FABP4 proved adipogenic differentiation. For chondrogenic differentiated cell pellets, the weak Alcian Blue staining was strengthened by strongly increased aggrecan, Col-II and COMP expression. With upregulation of important osteogenic markers like Runx2 and ALPL differentiation towards an osteogenic direction was underlined. The expression of Runx2 was not statistically significant since only n = 4 differentiations were performed for validation. It would be expected to find significant differences with more TLC cultures used. Furthermore, a longer differentiation of the cells as done for validation of adipogenic and chondrogenic differentiation could have improved the Runx2 expression. The early osteogenic marker osteocalcin was not increased in differentiated cells, which may be due to the fact that a 1 week time point was already too late. Furthermore the investigated alkaline phosphatase staining served as an additional control for a differentiation into the osteogenic direction. In the present study, the osteogenic differentiation potential was stronger in the female cells compared to the male cells from the previous study regarding the quantitative Alizarin Red S staining (OD: 0.08 versus 0.05; p = 0.007). A similar effect was seen in a study by Leskelä et al., who described an increasing osteogenic differentiation in bone marrow MSCs of women with age, but an unchanged differentiation rate in cells of male donors with age [41].

The cellular basis of sex-related differences is still a topic of controversy, when reviewing the current literature. The most likely factor causing differences between cells of men and women seems to be sex hormones such as estradiol. It has been reported that tenocytes of the posterior tibial tendon and flexor digitorum longus tendon of male and female donors express estrogen receptors, which are activated by estrogens such as estradiol [42]. Moreover, it has been found that estradiol had an effect on tendon fibroblasts by increasing Col-III and elastin expression [43], and inhibiting proliferation, and Col-I synthesis [44], when the hormone was directly added to the cell cultures. The influence of estrogens has also been shown in vivo, with the finding that collagen synthesis decreases in women with a higher hormone status [12], [45]. Despite these findings, the menstrual cycle seems to have no impact on tendon mechanical properties [46], [47], or collagen synthesis [12]. The present study was not able to prove a relationship between patients’ specific hormone levels and the cell biological findings presented. Sex hormones may not play a major role beyond the in vivo situation, when they are not directly added to the cells. However, it has to be kept in mind that the FCS in the medium contains an undefined amount of hormones and also phenol red, which is present in cell culture medium, representing a weakly acting estrogen mimic [48]. Since tenocytes of male and female donors were shown to express estrogen receptors [42], both components may influence TLCs in the same manner.

It has been suggested that the negative association linking female sex to inferior rotator cuff healing [9], [10] may, in addition to hormone levels, be due to differences in working activities and/or general daily activities between men and women [13], [14]. However, we were unable to investigate these associations as we were not provided with any additional information regarding our donors’ working activities or activities of daily living.

The cell count, Col-I expression, and protein synthesis, which seem to be important factors for the tendon bone healing of the rotator cuff, were stimulated in cells of female donors younger or older than 65 years of age with application of BMP-2 and BMP-7. These results are in accordance with several studies, which have reported that BMP-2 and BMP-7 have a positive effect on tendon and ligament cell cultures [16]–[21]. As observed in earlier studies [15], [16], BMP-7 had a stronger stimulating effect on the cells than BMP-2. The varying effect of BMP-2 and BMP-7 on the TLCs may be due to their different molecular structure and the different binding affinities of the molecules to BMP and activin receptors [49], [50]. BMP-7 binds with high affinity to the activin receptors I, II and IIB, whereas BMP-2 binds with high affinities to the BMP receptors IA, IB and II [51], [52]. We have previously shown that TLCs express BMP receptor IA and II, as well as activin receptor I and II, but only slightly express BMP receptor IB and activin receptor IIB [16]. However, we cannot draw any conclusions from the previous findings of receptor expression in TLCs, as they relate to the different stimulation capacities of BMP-2 and -7 seen in the present findings, since the needed receptors for both BMPs are expressed on the cells. However, when BMP-2 and BMP-7 were tested on C2C12 cells by osteoinduction (ALP activity) it was shown that both factors have the same relative activity at a concentration of 1000 ng/ml in this cell line and a higher relative activity of BMP-2 compared to BMP-7 was observed at a concentration of 200 ng/ml. This indicates that TLCs may not be able to process BMP-2 as good as BMP-7. Further studies are necessary to elucidate BMP/activin receptors and their ligands within this TLC population in more detail.

The important cellular biological characteristics can be augmented in TLCs of female donors younger and older than 65 years of age. Interestingly, however, cells of older female donors had a weaker stimulation potential with respect to cell count compared to TLCs of younger females and of older males from the previous study. The stimulation of cell count was slightly increased in the younger female and older male group [15] compared to the control. But the TLCs of females older than 65 years of age showed a slightly decrease in cell count compared to the untreated control. The stimulation of cell count was significantly decreased in the older female group compared to the other groups (p = 0.034–0.004), primarily at the earlier time points of days 3 and 5. We therefore speculate that the cell response to the growth factors takes longer in the cells of females older than 65 years of age, because of a slower cell metabolism, as already discussed by the biological characteristics of the cells.

In the previous study no distinct age-related differences for the stimulation potential in young and aged male donors were observed [15]. In general, it appears that the clinical parameters of older age in combination with the female sex have a negative influence on the stimulation potential of TLCs compared to other donors groups. Possibly, a higher dosage of growth factors would be needed to obtain the same effect in cells of older female donors compared to other donor groups. In vivo animal models could be used to identify the effect and the optimal dosage of BMP-2 and BMP-7 in different age and sex groups. For the potential future clinical application of BMP-2 or BMP-7 in rotator cuff repair, a higher dose for the treatment of older females should be considered.

### Conclusion

The present study revealed differences in the cell biological characteristics between different patient groups. From the present findings we suggest that inferior rotator cuff healing, which is often present in donors older than 65 years of age or females, is possibly associated with inferior cellular biological characteristics, which we have observed in TLCs of older female donors. A slower cell metabolism may also have an impact on the stimulation potential of TLCs.

We conclude from the present findings that for treatment options, such as growth factor application, the patients’ clinical characteristics should be considered in order to allow for more personalized therapy.
