# Correction: Characteristics and Stimulation Potential with BMP-2 and BMP-7 of Tenocyte-Like Cells Isolated from the Rotator Cuff of Female Donors

**DOI:** 10.1371/annotation/d6f80aa3-2ad7-4bf1-a9ff-426b2739b1c6

**Published:** 2013-10-01

**Authors:** Franka Klatte-Schulz, Stephan Pauly, Markus Scheibel, Stefan Greiner, Christian Gerhardt, Jelka Hartwig, Gerhard Schmidmaier, Britt Wildemann

A funding organization was incorrectly omitted from the Funding Statement. The first sentence of the Funding Statement should read: "The study was financially supported by the Deutsche Vereinigung für Schulter- und Ellenbogenchirurgie (DVSE, a non-profit organization, http://www.dvse.info/) and the European Society for Surgery of the Shoulder and the Elbow (SECEC/ESSSE, a non-profit organization, http://www.secec.org/)."

